# Emergence of outbreak-driving high-risk Pseudomonas aeruginosa lineages in Taiwan: phylogenomic insights into ST292 and the ST235* sublineage

**DOI:** 10.1099/mgen.0.001694

**Published:** 2026-04-21

**Authors:** Chih-Ming Chen, Hui-Ling Tang, Se-Chin Ke, Yi-Pei Lin, Bo-Han Chen, Ru-Hsiou Teng, Chien-Shun Chiou, Min-Chi Lu, Yi-Chyi Lai

**Affiliations:** 1 Department of Internal Medicine, Tungs’ Taichung MetroHarbor Hospital, Taichung, Taiwan, ROC; 2 College of Medicine, National Chung Hsing University, Taichung, Taiwan, ROC; 3 Department of Microbiology and Immunology, School of Medicine, China Medical University, Taichung, Taiwan, ROC; 4 Infection Control Office, Tungs’ Taichung MetroHarbor Hospital, Taichung, Taiwan, ROC; 5 Department of Medical Technology, Jen-Teh Junior College of Medicine, Nursing and Management, Miaoli, Taiwan, ROC; 6 Department of Medical Research, Tungs’ Taichung MetroHarbor Hospital, Taichung, Taiwan, ROC; 7 Central Region Laboratory, Center for Diagnostics and Vaccine Development, Centers for Disease Control, Ministry of Health and Welfare, Taipei, Taiwan, ROC; 8 Division of Infectious Diseases, Department of Internal Medicine, China Medical University Hospital, Taichung, Taiwan, ROC; 9 Department of Microbiology and Immunology, School of Medicine, Chung Shan Medical University, Taichung, Taiwan, ROC; 10 Department of Internal Medicine, Chung Shan Medical University Hospital, Taichung, Taiwan, ROC

**Keywords:** carbapenem-resistant (CR), extensively drug-resistant (XDR), *Pseudomonas aeruginosa*, ST235*, ST292, VIM (Verona Integron-encoded Metallo-Beta-lactamase)-producing ST244

## Abstract

The global rise of carbapenem-resistant *Pseudomonas aeruginosa* (CRPA) poses a significant threat to public health. Through active surveillance conducted between 2021 and 2022, we collected 92 distinct CRPA isolates and identified a clonal shift toward high-risk lineages ST235* and ST292. Whole-genome sequencing and phylogenomic analysis revealed that ST235*, a single-locus variant of epidemic ST235, exhibited broader antimicrobial resistance (AMR) and carried a *nalD* loss-of-function mutation, resulting in MexAB-OprM overexpression. Two ST235* isolates contained a hybrid resistance cassette with *bla*_KPC-2_ and *bla*_TEM-1_, likely acquired through IS*26*-mediated transfer from *Enterobacter hormaechei*. Most ST292 isolates demonstrated an extensively drug-resistant phenotype with a *bla*_CARB-2_-containing cassette and a chromosomally integrated tMexC3D-OprJ efflux system. Additionally, ST244 isolates possessed a *bla*_VIM-2_ cassette embedded within a Tn*402* transposition module (t*niABQR-intI1*). Multiple copies of *tniABQR-intI1* carrying AMR genes were exclusively found in the genomes of ST244, representing a novel mechanism. The genomic plasticity of high-risk CRPA lineages emphasizes their capacity to acquire, amplify and disseminate AMR determinants. To combat the spread of CRPA, genomic surveillance and targeted infection control strategies are urgently required.

**Impact Statement**

This study provides crucial genomic insights into the emergence and evolution of high-risk CRPA lineages, ST235*, ST292 and VIM (Verona Integron-encoded Metallo-β-lactamase)-producing ST244, leading to extensive drug resistance beyond carbapenems in clinical settings. Genomic features, including a *nalD* loss-of-function mutation in ST235*, a chromosomally integrated *tMexCD-OprJ* with the acquisition of *bla*_CARB-2_ cassette in ST292, and Tn*402*-mediated transposition of *bla*_VIM-2_ cassette in ST244, emphasize the genomic plasticity of CRPA and its capacity for rapid adaptation and dissemination. Localized genomic surveillance and tailored infection control strategies are urgently needed.

## Data Summary

The genome sequencing data for the *P. aeruginosa* strains analysed in this study are publicly available in GenBank under BioProject PRJNA1266355. Accession numbers for individual strains are listed in Table S1, available in the online Supplementary Material. This article includes all data generated or analysed during this study. The corresponding authors will provide any additional information upon reasonable request.

## Introduction

*Pseudomonas aeruginosa* is a highly adaptable Gram-negative pathogen and a leading cause of healthcare-associated infections, including ventilator-associated pneumonia, bacteraemia, urinary tract infections and surgical site infections [1]. The emergence and dissemination of carbapenem-resistant *P. aeruginosa* (CRPA) significantly limit treatment options [2]. The urgent need for enhanced surveillance for CRPA is emphasized by the statement that the World Health Organization has classified CRPA as a high priority pathogen. Adding to this threat is the increasing prevalence of extensively drug-resistant (XDR) and difficult-to-treat (DTR) strains of *P. aeruginosa*. XDR is defined as non-susceptibility to at least one agent in all but two or fewer antimicrobial categories, while DTR refers to isolates nonsusceptible to all first-line agents, including *β*-lactams (e.g. piperacillin-tazobactam, ceftazidime, cefepime and aztreonam) and fluoroquinolones. XDR and DTR *P. aeruginosa* have risen to prominence in intensive care units (ICUs) and patients with underlying comorbidities or prior exposure to broad-spectrum antibiotics and are associated with prolonged hospital stays, elevated healthcare costs and worsened survival outcomes [3].

Antimicrobial resistance (AMR) in *P. aeruginosa* arises through various mechanisms, including target site mutations, porin loss, multidrug efflux pump overexpression and AMR gene acquisition. The global increase of CRPA is primarily attributed to the dissemination of high-risk clones, notably sequence types (STs) ST235, ST111, ST175, ST244, ST308 and ST654. ST235 is the most extensively distributed lineage and is frequently associated with chromosomal mutations and carriage of carbapenemase genes such as *bla*_VIM_ and *bla*_IMP_, contributing to outbreaks across Europe, Asia and South America [4]. In Israel, CRPA bloodstream infections have been dominated by *bla*_GES_-producing ST654 [5], while ST175 and ST244 are commonly reported in Spain [6]. In Southeast Asia, ST308 and ST235 carrying the *bla*_NDM-1_ gene have been implicated in sporadic infections and nosocomial outbreaks in Singapore [7]. These high-risk clones often co-harbour genes coding for carbapenemases, virulence and mobile genetic elements that enhance their adaptability, persistence and global transmission potential.

Over the past decade, CRPA has become a growing clinical challenge in Taiwan. Data from national surveillance programmes, such as SMART and TSAR, have shown a steady increase in CRPA prevalence, rising from 12.3% in 2012 to 22.8% in 2020, with particularly high rates reported in the northern region [8]. Despite the rising prevalence, no comprehensive studies have been conducted in Taiwan to date that analyse the genomic features and phylogenomic relatedness of CRPA, particularly concerning high-risk lineages. In response to this gap, we performed whole-genome sequencing (WGS) of CRPA isolates collected between 2021 and 2022. We elucidated the genomic features, resistance mechanisms and clonal relatedness of CRPA lineages circulating in a hospital. Our work provides the first genomic framework for CRPA epidemiology, offering critical baseline data to support national surveillance, infection control and antimicrobial stewardship initiatives.

## Methods

### CRPA isolates

Between July 2021 and September 2022, a total of 92 non-duplicate CRPA isolates were collected from patients admitted to Tungs’ Taichung MetroHarbor Hospital, a regional teaching hospital comprising 24 clinical departments and 1,381 beds. In addition, two carbapenem-intermediate (carbapenem-intermediate *P. aeruginosa*, CIPA) and two carbapenem-susceptible isolates (carbapenem-susceptible *P. aeruginosa*, CSPA) were included as controls for comparative analysis. Bacterial species identification was performed using the Bruker MALDI Biotyper^™^ system (Bruker Daltonics, Germany). Antimicrobial susceptibility testing (AST) was conducted using the Phoenix Automated Microbiology System (BD Diagnostics, Sparks, MD, USA). MICs for imipenem and meropenem were determined using the agar dilution method, while aztreonam susceptibility was assessed via the disc diffusion method (30 µg; Liofilchem, Italy). All susceptibility results were interpreted according to the Clinical and Laboratory Standards Institute (CLSI) breakpoints, as specified in M100-ED23. Quality control was ensured by using standard reference strains recommended by CLSI.

### Pulsed-field gel electrophoresis

The clonal relatedness of CRPA isolates was assessed using standardized PFGE with the CHEF-DR III system (Bio-Rad Laboratories Inc., USA). *Xba*I-digested genomic DNA fragments were analysed using GelCompar II software (version 6.5, Applied Maths, Belgium). The similarity between banding patterns was calculated using the Dice coefficient, and clustering was performed using the unweighted pair group method with arithmetic mean, applying a 1% optimization and 1% position tolerance. Genomic DNA from *Salmonella enterica* Braenderup H9812, digested with *Xba*I, was used as the molecular size marker. The resulting dendrogram defined PFGE clusters based on a similarity threshold of ≥80%.

### WGS, genome profiling and comparative genomic analysis

Genomic DNA was extracted from 43 representative CRPA isolates. All isolates were subjected to short-read sequencing on the Illumina MiSeq platform (Illumina, San Diego, USA) using the Illumina DNA Prep kit. To facilitate high-quality assembly, a subset of 37 strains was additionally sequenced on the Nanopore MinION platform (Oxford Nanopore Technologies, Oxford, UK). Long-read libraries were prepared using the Rapid Barcoding Kit 96 (SQK-RBK110.96) and loaded onto R9.4.1 flow cells (FLO-MIN106D), following the manufacturer’s standard protocols. Chromosomal assembly was performed using Flye *v*2.9.4, followed by long-read polishing with Medaka *v*1.11.3. To improve consensus accuracy, assemblies were further polished with short reads using pypolca *v*0.3.0. Plasmid assembly was performed using Plassembler *v*1.6.1. To ensure data quality, assemblies were filtered to remove contigs smaller than 1,000 bp or with coverage <20×. All software tools were executed using default parameters unless otherwise specified. The assembled genomes are publicly available in GenBank under BioProject PRJNA1266355 (Table S1). Genome annotation was performed using the Rapid Annotations using Subsystems Technology server (https://rast.nmpdr.org/). AMR genes were identified using ResFinder through the Centre for Genomic Epidemiology (http://www.genomicepidemiology.org/) and the Comprehensive Antibiotic Resistance Database (CARD) (https://card.mcmaster.ca/). Comparative genomic analyses were performed using Geneious Prime 2025.1.2 (Biomatters, New Zealand).

### Core-genome SNP analysis

To assess local diversity, whole-genome assemblies of 43 representative *P. aeruginosa* strains were analysed for core-genome SNPs (cgSNPs) using Parsnp *v*1.2 with default parameters. A maximum-likelihood phylogenetic tree was constructed from SNP calls in core-genome alignments, using *P. aeruginosa* strain KB-PA_F19 [9] (ST244; CP086010.1) as the reference. The resulting cgSNP-based phylogenetic tree was visualized using Geneious Prime and annotated with relevant metadata, including the date of isolation, ST, resistome, resistance-nodulation-cell division (RND) efflux pump systems, mutations in OprD, ParC and GyrA and phenotypic AST results. To contextualize the ST235 isolates globally, a separate phylogenomic analysis was performed on 131 ST235 genomes, comprising 128 sequences retrieved from the NCBI Pathogen Detection database and the 3 isolates in this study. This dataset was analysed with Gubbins v3.4.3 to remove recombination regions and reconstruct the phylogeny, which was then visualized in iTOL *v*7.4.2.

## Results

### Emergence of high-risk clones of CRPA

**Fig. 1.**
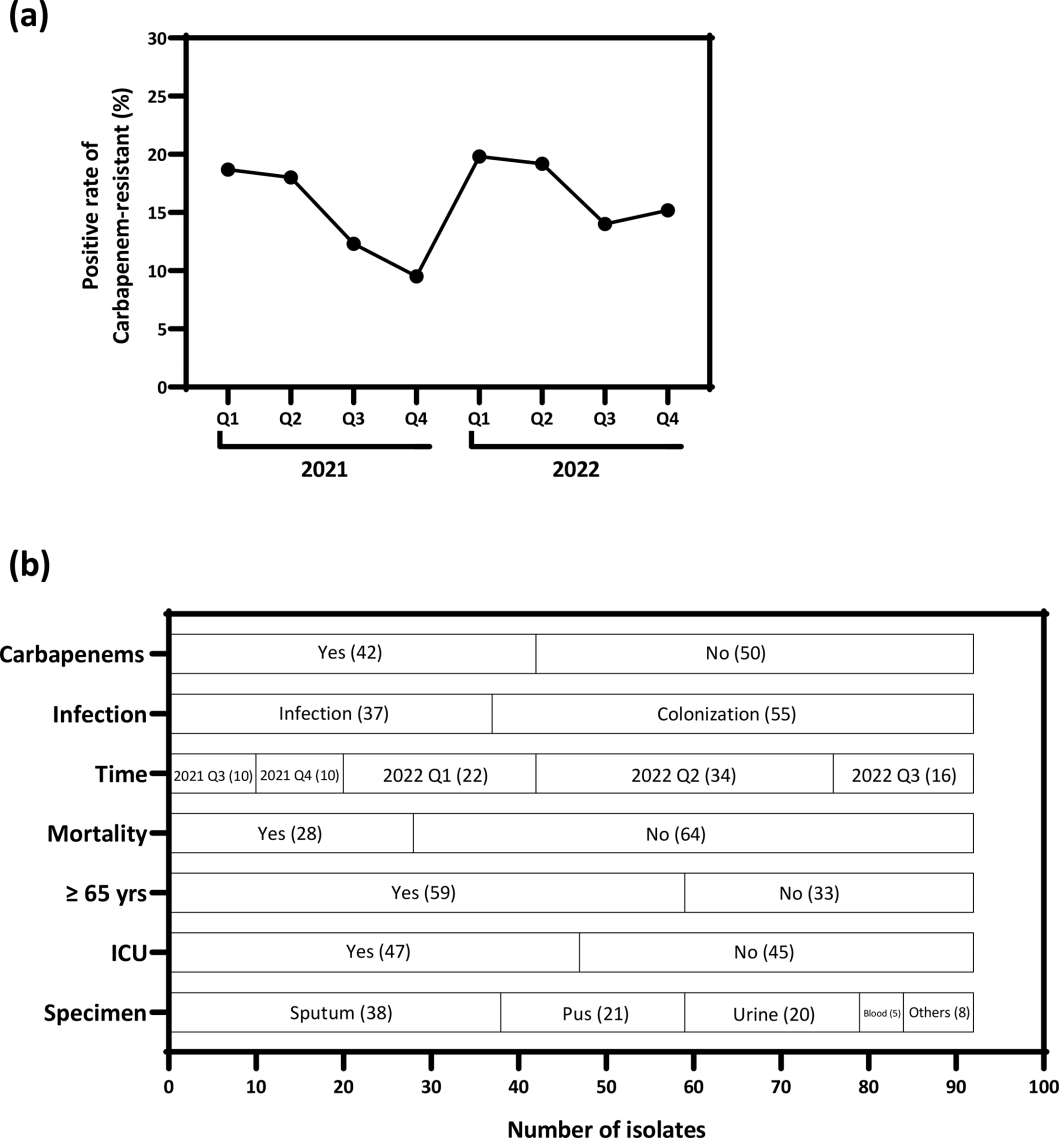
CRPA surveillance and clinical characteristics. (**a**) The carbapenem resistance rates among *P. aeruginosa* isolates in our hospital ranged from 9.5% to 19.8% during 2021–2022. (**b**) A total of 92 CRPA isolates were collected for analysis. Demographic and clinical characteristics of the corresponding patients are summarized, including specimen type, patient age, infection versus colonization status, 30-day mortality, ICU admission and prior exposure to carbapenems.

We conducted continuous active surveillance of multidrug-resistant *P. aeruginosa* to monitor trends in AMR. Between 2021 and 2022, the detection rate of CRPA ranged from 9.5% to 19.8% (Fig. 1a). Although these rates were slightly lower than the nationwide average in Taiwan, which exceeded 20% [10,11], a marked surge in CRPA cases was observed during the first and second quarters of 2022. To investigate whether the emergence of high-risk clones contributed to this increase, we collected all available non-duplicate CRPA isolates obtained between July 2021 and September 2022 and performed comprehensive phylogenetic analyses to evaluate clonal relatedness. In total, 92 CRPA isolates were included in the study, along with two CIPA and two CSPA isolates as controls. Sputum was the most common specimen source for CRPA isolation (38 out of 92; 41.3%). Most CRPA strains were isolated from individuals aged ≥65 years (64.1%) and those admitted to ICUs (51.1%). Additionally, 45.7% of the CRPA isolates were from patients with documented prior carbapenem exposure (Fig. 1b).

**Fig. 2.**
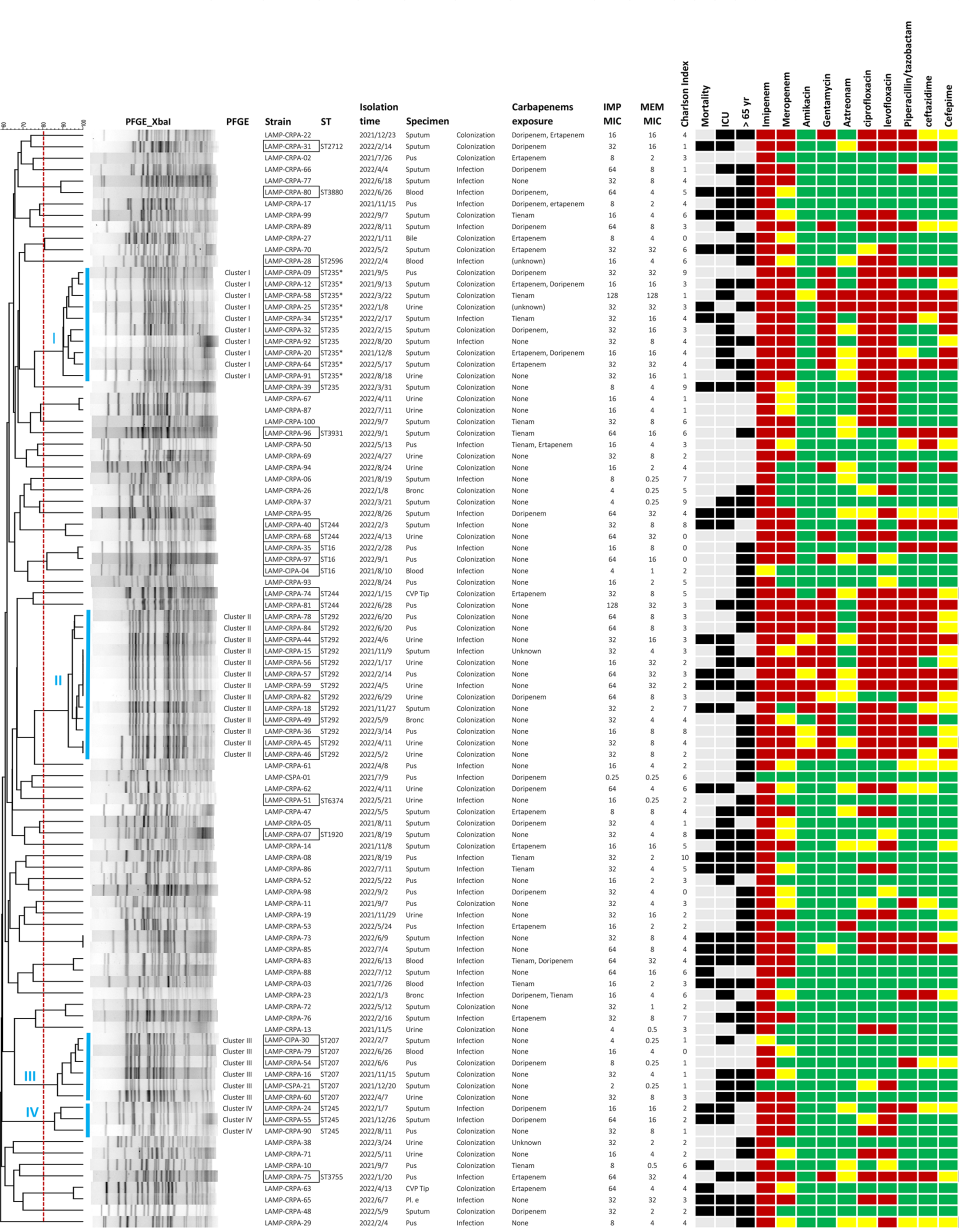
PFGE profiling and clinical metadata of CRPA isolates (*n*=92) collected from July 2021 to September 2022. Isolates were grouped based on *Xba*I-digested PFGE profiles. For each non-duplicate isolate, associated clinical and demographic data are presented, including date of isolation, specimen type, infection versus colonization, prior exposure to carbapenems, Charlson comorbidity index, 30-day mortality, ICU stay and whether the patient was aged ≥65 years. Antibiotic susceptibility testing results are shown in red (resistant), yellow (intermediate) and green (susceptible) colours. The presence or absence of selected clinical characteristics is indicated in black or grey. STs were determined using the *P. aeruginosa* MLST scheme through the Centre for Genomic Epidemiology (http://www.genomicepidemiology.org/). Isolates selected for WGS are outlined in black.

**Fig. 3.**
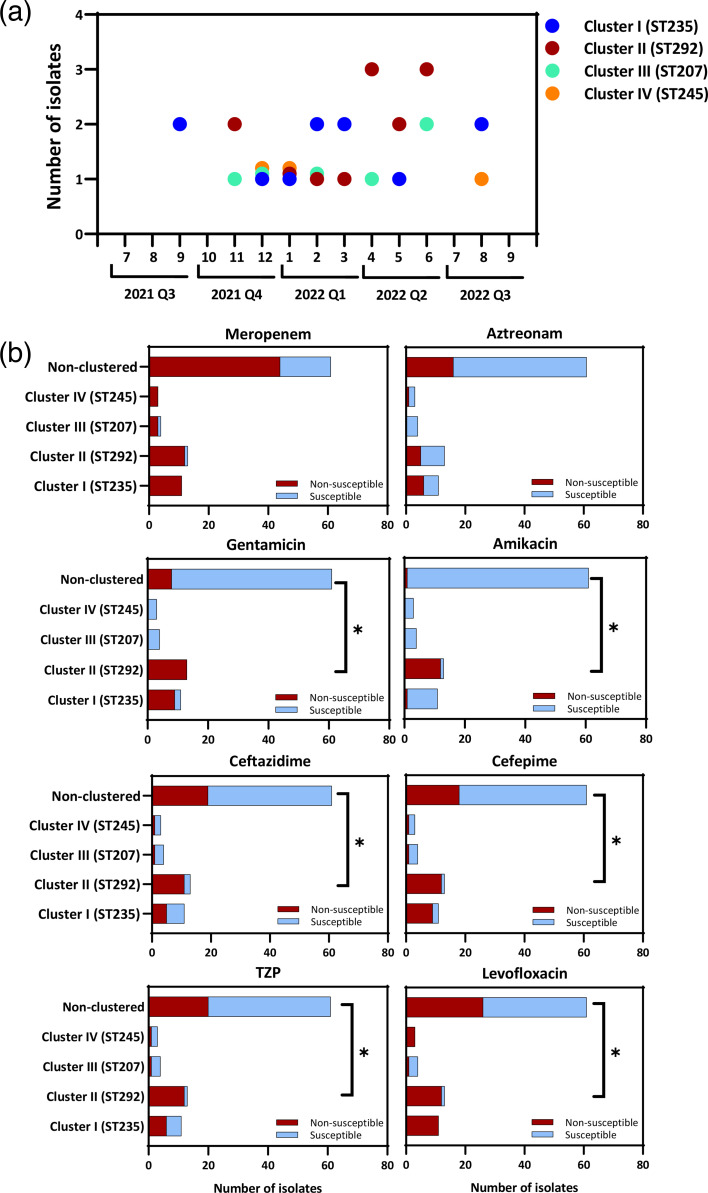
Temporal distribution and AMR profiles of CRPA isolates stratified by PFGE cluster. (**a**) The isolation timeline of CRPA isolates assigned to PFGE-defined clusters I–IV (≥80% similarity) is shown as blue, brown, green and orange dots, respectively. (**b**) All 92 CRPA isolates were non-susceptible to imipenem. The presence of non-susceptibility to additional antimicrobials, including meropenem, aztreonam, gentamicin, amikacin, ceftazidime, cefepime, piperacillin-tazobactam (TZP) and levofloxacin, is shown for each isolate, grouped by PFGE cluster (I, II, III, IV) and a non-clustered group. Associations between PFGE cluster and antimicrobial non-susceptibility were assessed using the Pearson chi-square or Fisher’s exact test if frequencies were <5. A two-tailed *P*-value<0.05 was considered statistically significant.

Although the overall genetic background of the CRPA isolates demonstrated substantial diversity, PFGE analysis identified four distinct clusters defined by ≥80% similarity. To facilitate epidemiological interpretation, we integrated MLST data to link these clusters to specific genomic lineages. The identified clusters were as follows: Cluster I (*n*=11), corresponding to the ST235 lineage (including the ST235 variant); Cluster II (*n*=13), corresponding to ST292; Cluster III (*n*=4) to ST207; and Cluster IV (*n*=3) to ST245 (Fig. 2). The first isolate of Cluster I (ST235/235*) was detected in September 2021. Subsequently, isolates belonging to Clusters II (ST292), III (ST207) and IV (ST245) were detected in the fourth quarter of 2021. The continued identification of isolates from all four clusters through the second quarter of 2022 (Fig. 3a) indicates a temporal increase, suggesting potential intra-hospital persistence during this period. Notably, CRPA isolates in Clusters I (ST235/235*) and II (ST292) frequently exhibited co-resistance to multiple classes of antimicrobials, including aminoglycosides (gentamicin and amikacin), extended-spectrum cephalosporins (ceftazidime and cefepime) and fluoroquinolones (levofloxacin) (Fig. 3b). Based on the temporal persistence and multidrug resistance profiles, Clusters I (ST235/235*) and II (ST292) were considered potential high-risk clones of CRPA.

### Phylogenomic relatedness and intrinsic resistome of CRPA strains

**Fig. 4.**
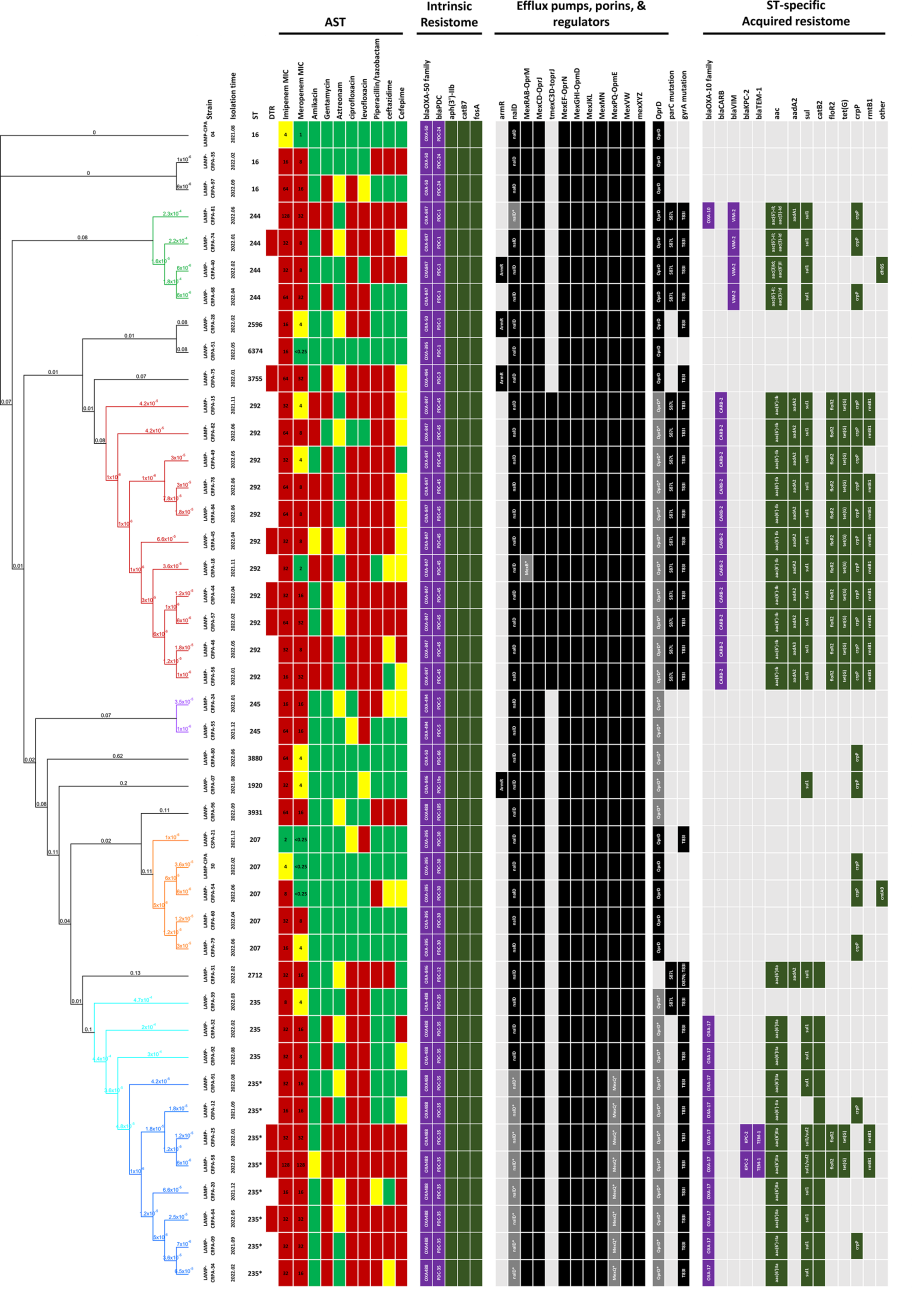
Phylogenomic relatedness and resistome profiles of high-risk *P. aeruginosa* lineages. Whole-genome assemblies of 43 representative *P. aeruginosa* isolates were analysed using cgSNP analysis performed with Parsnp v1.2. A maximum-likelihood phylogenetic tree was generated using *P. aeruginosa* KB-PA_F19 (CP086010.1) as the reference genome. The resulting cgSNP-based phylogeny was visualized and annotated in Geneious Prime, incorporating metadata such as isolation date, ST, resistome composition and phenotypic AST results. The distance between nodes is presented as the substitution rate per site. The presence of AMR genes, identified using ResFinder (Centre for Genomic Epidemiology; http://www.genomicepidemiology.org/), is indicated in black or designated colours. Absence of AMR genes is shown in light grey.

To investigate the genomic features of potential high-risk clones, we selected representative *P. aeruginosa* strains from PFGE clusters I–IV, along with a subset of genetically diverse non-clustered strains, for WGS. After quality control and removal of low-quality assemblies, 43 genome assemblies were subjected to further analysis. cgSNP analysis was performed using Parsnp *v*1.2, with *P. aeruginosa* KB-PA_F19 (ST244; CP086010.1) as the reference (Fig. 4). MLST, based on the seven housekeeping genes (*acsA*, *aroE*, *guaA*, *mutL*, *nuoD*, *ppsA* and *trpE*), confirmed the concordance between PFGE clusters and STs described above. Among the non-clustered strains, the following STs were identified: ST16 (*n*=3), ST244 (*n*=4), ST2596 (*n*=1), ST3755 (*n*=1), ST3880 (*n*=1), ST1920 (*n*=1), ST3931 (*n*=1), and ST6374.

The resistome of each strain was characterized by integrating predictions from ResFinder and CARD. The intrinsic resistome, shared by all CRPA strains, included chromosomally encoded genes: *bla*_OXA_ and *bla*_PDC_ (*β*-lactamases), *aph(3’)-IIb* (aminoglycoside-modifying enzyme), *catB7* (chloramphenicol acetyltransferase) and *fosA* (glutathione S-transferase). These genes conferred resistance to penicillin and certain cephalosporins, streptomycin and spectinomycin, chloramphenicol and fosfomycin, respectively (Fig. S1). The intrinsic *bla*_OXA_ subtypes, which correlated with respective STs, were all variants of the *bla*_OXA-50_ family and are known to exhibit weak hydrolytic activity. Similarly, the *bla*_PDC_ variants, also ST-specific, encoded the AmpC-type *β*-lactamases (Fig. S2a, b), contributing to intrinsic resistance to penicillin and first- and second-generation cephalosporins. Most CRPA strains harboured a complete repertoire of major RND family efflux systems, including MexRAB-OprM, MexCD-OprJ, MexEF-OprN, MexGHI-OpmD, MexJKL, MexMN, MexPQ-OpmE, MexVW and MexXYZ (Fig. S3). In addition, the amino acid sequences of the orphan porin OprD shared 86% pairwise identity with the reference *P. aeruginosa* PAO1 OprD protein. Notably, premature stop codons in *oprD* were frequently observed, particularly in strains of ST292 and the ST235* sub-lineage (Fig. S2c, d), potentially contributing to carbapenem resistance. While the anti-repressor ArmR, which neutralizes MexR repression of *mexAB*, was detected in only a few isolates, NalD, another transcriptional repressor of *mexAB*, harboured a premature stop codon exclusively in the ST235* sub-lineage. Genomically, this loss-of-function mutation is predicted to lead to the constitutive derepression of the MexAB efflux pump in this high-risk sublineage.

### Emergence of a high-risk sub-lineage of ST235 CRPA, ST235*

**Fig. 5.**
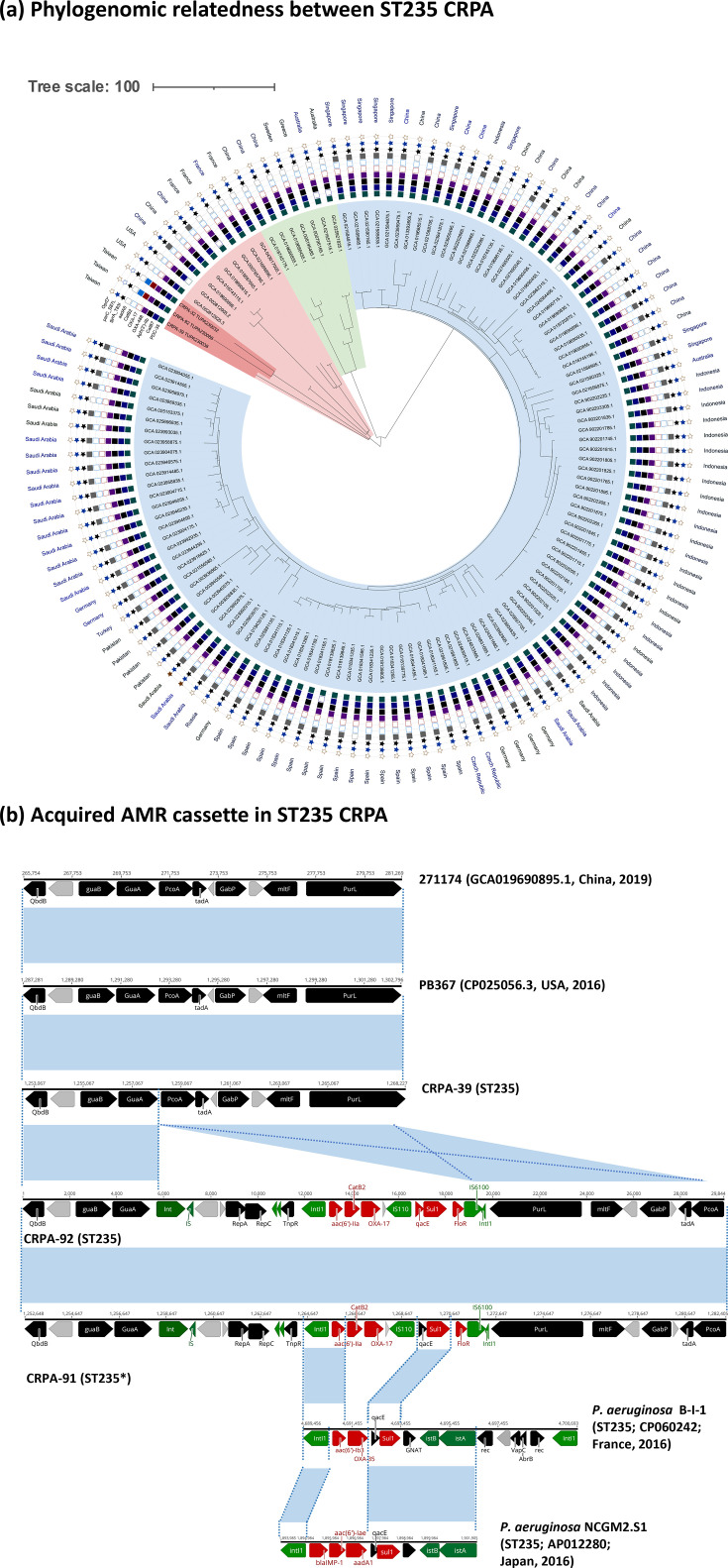
Comparative genomic analysis of ST235 CRPA isolates. (**a**) Circle cgSNP phylogeny of ST235 CRPA. A total of 128 genomes from the NCBI Pathogen Detection database and 3 from this study were analysed using Gubbins (*v*3.4.3) and visualized in iTOL *v*7.4.2. The tree highlights major sublineages shaded in pink (**i**), light green (ii) and light blue (iii), with isolates in this study marked in dark red. Concentric rings indicate the presence of AMR genes (*bla*_PDC-35_, *catB7*, *aph(3')-IIb*, *bla*_OXA-488_, *bla*_OXA-17_, *catB2* and *aadA6*) and chromosomal mutations (gyrA_T83I, parC_S87L and *oprD*), with geographic origins labelled at the perimeter; the scale bar represents 100 SNPs. (**b**) Comparative analysis of acquired AMR cassettes between our ST235 and ST235* CRPA isolates and related strains: *P. aeruginosa* PB367 (CP025056.3, USA, 2016), 271174 (GCA_019690895.1, China, 2019), B-I-1 (CP060242.1, France, 2016) and NGGM2.S1 (AP012280.1, Japan, 2016). Differences in gene content and cassette architecture are highlighted.

The ST235 lineage of *P. aeruginosa* is globally recognized as a high-risk clone. This study identified three ST235 strains and eight strains belonging to a distinct sub-lineage within ST235, designated ST235* (38-novel-3-13-1-2-4), characterized by a unique variant of the *aroE* allele and distinct cgSNP signatures (Fig. 4). Both ST235 and ST235* strains carried *exoU*, encoding a type III secretion system effector protein associated with increased cytotoxicity and severe clinical outcomes [12]. Notably, *exoU* was absent in all other STs of CRPA strains analysed in this study. To contextualize our isolates within a global phylogenomic framework, we analysed 128 publicly available ST235 genomes retrieved from the NCBI Pathogen Detection database, along with our 3 study strains. A cgSNP phylogeny was reconstructed using Gubbins (*v*3.4.3) and visualized with iTOL *v*7.4.2 (Fig. 5a). The resulting tree resolved the global ST235 population into three sublineages (I, II and III). Our local ST235 isolates clustered within sublineage III and were most closely related to strains PB367 (GCA_002812925.3, USA, 2016), 271174 (GCA_019690895.1, China, 2019) and B-I-1 (GCA_15697645, France, 2016). Resistome analysis identified heterogeneity in acquired resistance determinants. CRPA-39, which exhibited intermediate meropenem resistance (MIC=4 µg ml^−1^), carried only the intrinsic resistome, *bla*_OXA-488_, *bla*_PDC_, *aph(3')-IIb*, *fosA* and *catB7*, similar to PB367 (USA, 2016) and 271174 (China, 2019) (Fig. 5b). In contrast, CRPA-32 and CRPA-91 carried additional resistance genes [*bla*_OXA-17_, *aac(6')-IIa*, *sul1* and *catB2*], within class I integrons. Integron gene cassette composition varied among related global isolates. For instance, the B-I-1 strain from France carried *bla*_OXA-35_, *aac(6')-Ib3* and *sul1*, while the NCGM2.S1 [13] strain from Japan contained *bla*_IMP-1_, *aac(6')-Iae* and *aadA1* (Fig. 5b).

**Fig. 6.**
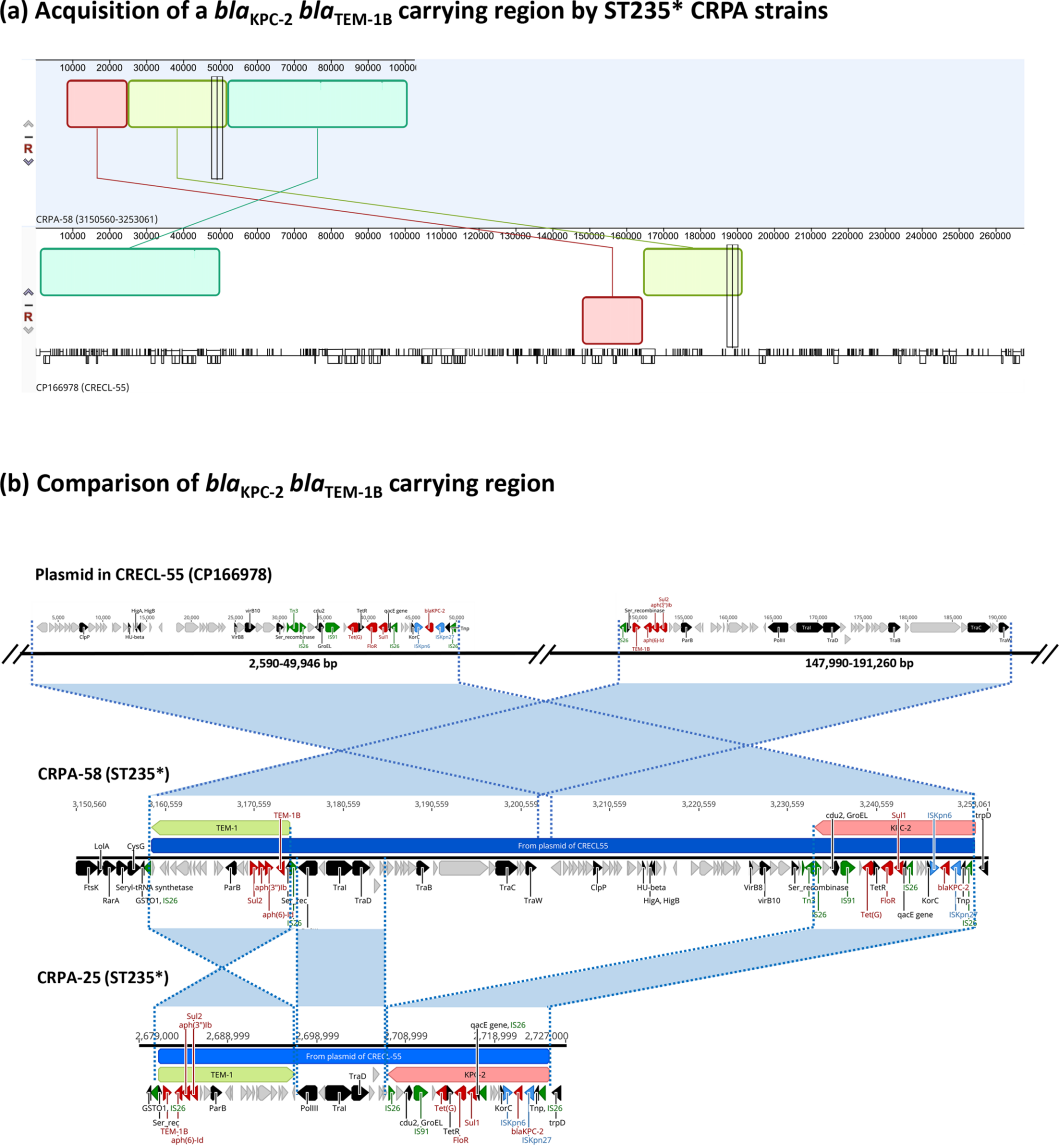
Acquisition of a *bla*_KPC-2_- and *bla*_TEM-1_-containing hybrid region in ST235 CRPA. (**a**) Mauve alignment of the *bla*_KPC-2_–*bla*_TEM-1_ region in CRPA-58 with a homologous region from a plasmid in *E. hormaechei* CRECL-55 (CP166978). (**b**) Comparative analysis of the *bla*_KPC-2_–*bla*_TEM-1_ regions in CRPA-58 and CRPA-25 with the corresponding region in the CRECL-55 plasmid, highlighting conserved blocks and variations.

Eight strains exhibiting a single-locus variant of ST235, designated as ST235*, were identified in this study. Similar to ST235, all ST235* strains harboured the acquired resistome [*bla*_OXA-17_, *aac(6')-IIa*, *sul1* and *catB2*] and displayed a defective form of the OprD porin due to a premature stop codon mutation. However, a unique premature stop codon in *nalD*, a repressor of *mexAB-oprM*, was found exclusively in the ST235* sub-lineage. This loss-of-function mutation likely contributed to MexAB overexpression, thereby enhancing broad-spectrum resistance. Moreover, two ST235* strains, CRPA-25 and CRPA-58, acquired additional AMR cassettes carrying *bla*_KPC-2_ and *bla*_TEM-1_. These cassettes, separated by *tra*-containing regions, shared a high degree of similarity with an IncC-IncFII hybrid plasmid identified in an ST78 *Enterobacter hormaechei* strain, CRECL-55 (CP166978) (Fig. 6a). A 47 kb region containing a *bla*_KPC-2_ cassette with the conserved core structure, identified as IS*Kpn6-bla*_KPC-2_-IS*Kpn27*, along with *sul2*, *floR* and *tet(G*), and a 43 kb region containing *bla*_TEM-1_, *aph(6)-Id*, *aph(3’)-Ib* and *sul2*, was jointly incorporated into the intergenic region of GSTO1-TrpD in CRPA-58 via IS*26*-mediated transposition. In contrast, CRPA-25 retained the core cassettes of *bla*_KPC-2_ and *bla*_TEM-1_ but lost a 47 kb fragment (Fig. 6b).

### Clonal expansion of ST292 *P. aeruginosa*

ST292 *P. aeruginosa* was first identified during an outbreak involving XDR strains in clinical settings in Kunming, China [14]. This resistance phenotype was primarily attributed to deletions in the *oprD* gene and overexpression of efflux pump systems [15]. Despite its clinical importance, genomic data on ST292 have remained limited. In this study, we performed WGS of ST292 isolates to further characterize the genomic features and phylogenomic relatedness of this high-risk lineage. To elucidate the global context, we incorporated six publicly available ST292 genomes from other countries into our analysis. The resulting cgSNP phylogenetic tree revealed that three of these international isolates clustered closely with our ST292 strains (Fig. S4), suggesting a high degree of genetic relatedness. Locally, as shown in Fig. 2, the ST292 strains (*n*=13) clustered within high-risk Cluster II based on PFGE profiling. Most ST292 CRPA strains exhibited an XDR phenotype, with extensive resistance to carbapenems and multiple other antimicrobial agents. Notably, three isolates, CRPA-44, CRPA-45 and CRPA-57, met the DTR criteria, strengthening the clinical significance of this ST292 lineage.

**Fig. 7.**
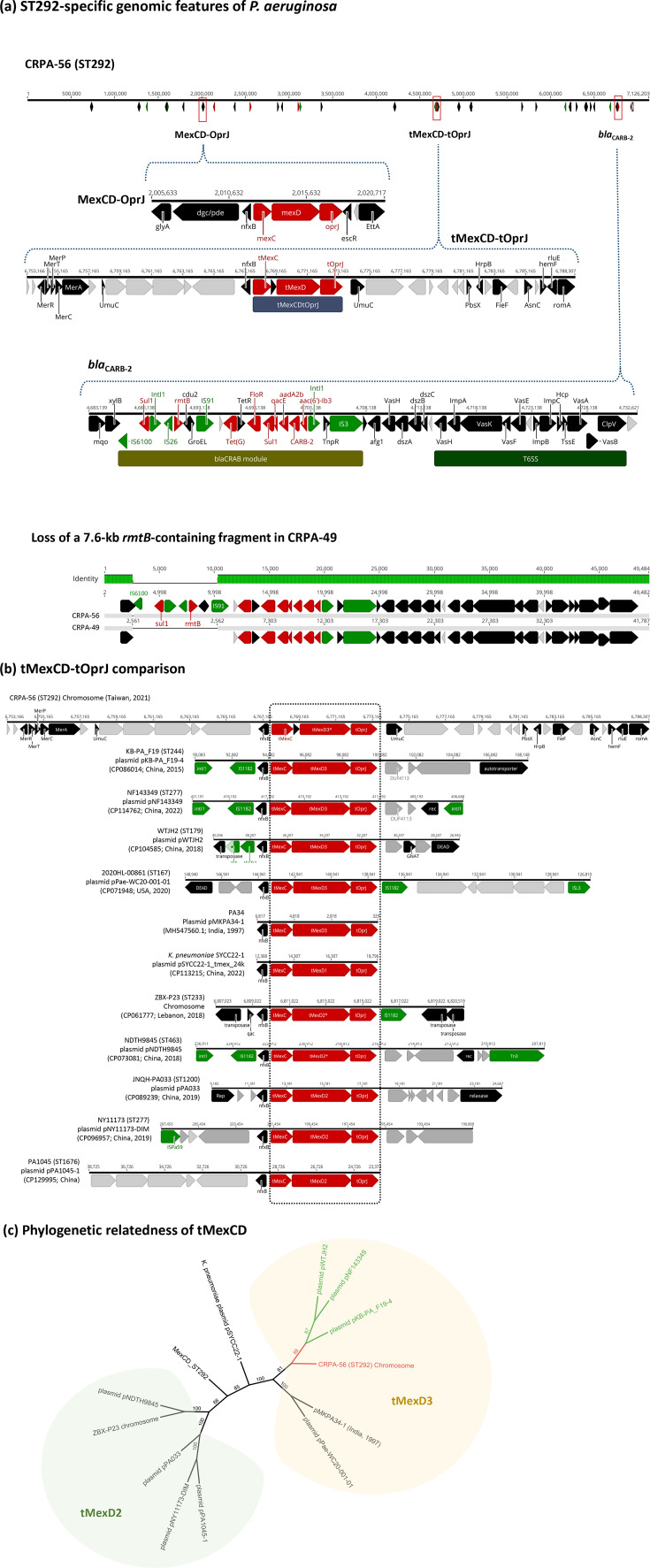
Genomic features of ST292 CRPA isolates. (**a**) In addition to acquiring a *bla*_CARB-2_-containing AMR cassette, all ST292 strains harboured a chromosomally integrated *tMexCD-OprJ* module – a plasmid-derived homologue of the intrinsic MexCD-OprJ efflux system. (**b**) Comparative analysis of the *tMexCD-OprJ* module from ST292 with plasmid-borne *tMexCD-OprJ* sequences identified in *P. aeruginosa* strains from various STs. (**c**) Phylogenetic analysis based on pairwise nucleotide sequence alignment of the *tmexCD-toprJ* loci. The *tmexD* sequence in ST292 was classified within the *tmexD3* cluster.

Beyond the intrinsic resistome, ST292 isolates carried a class I integron harbouring an AMR cassette that included *rmtB*, *tet(G*), *floR*, *sul1*, *aadA2b*, *blaCARB-2* and *aac(6’)-Ib3*. Except for CRPA-49, all ST292 isolates were non-susceptible to amikacin, likely due to the combined effects of *rmtB* and *aac(6’)-Ib3* (Fig. 7a). Although *bla_CARB-2_* primarily confers resistance to carboxypenicillins, its presence alongside *oprD* disruption may broaden resistance to additional *β*-lactams. All ST292 strains, except for CRPA-18 (which harboured a premature stop codon in *mexB*), demonstrated high-level resistance to imipenem (MIC₅₀=32 µg ml^−1^). Notably, all ST292 carried a chromosomally integrated *tMexCD-OprJ*, a plasmid-derived homologue of the chromosomal *MexCD-OprJ* efflux system. We retrieved *tMexCD-OprJ*-carrying plasmid sequences from complete *P. aeruginosa* genomes to explore their phylogenetic relatedness further. The plasmid-borne *tMexCD-OprJ* loci were among clinical *P. aeruginosa* strains with diverse genetic backgrounds (Fig. 7b). Unlike the *tMexCD-OprJ* found in *Klebsiella pneumoniae* plasmids [16], most *P. aeruginosa* sequences fell into the *tMexD2* or *tMexD3* subtypes (Fig. 7c). Compared with closely related plasmid-borne sequences, pKB-PA_F19-4 (CP086014), pNF143349 (CP114762) and pWTJH2 (CP104585), we identified three nonsynonymous mutations in ST292: an A-to-G transition in *tmexC*, a C-to-T transversion in *tmexD* and a C-to-G transition in *toprJ*, resulting in the amino acid changes tMexC (Trp235Ala), tMexD3 (Δ1–117) and tOprJ (Ala235Gly), respectively (Fig. S5a). The N-terminal deletion (Δ1–117) in tMexD3 likely disrupts a transmembrane domain and part of an extracellular motif (Fig. S5b). Given the role of *tMexCD-OprJ* in exporting a broad spectrum of structurally diverse antibiotics, this deletion may alter the function of the efflux system and further impact the multidrug resistance profile of ST292 strains.

### VIM-producing ST244 *P. aeruginosa*

**Fig. 8.**
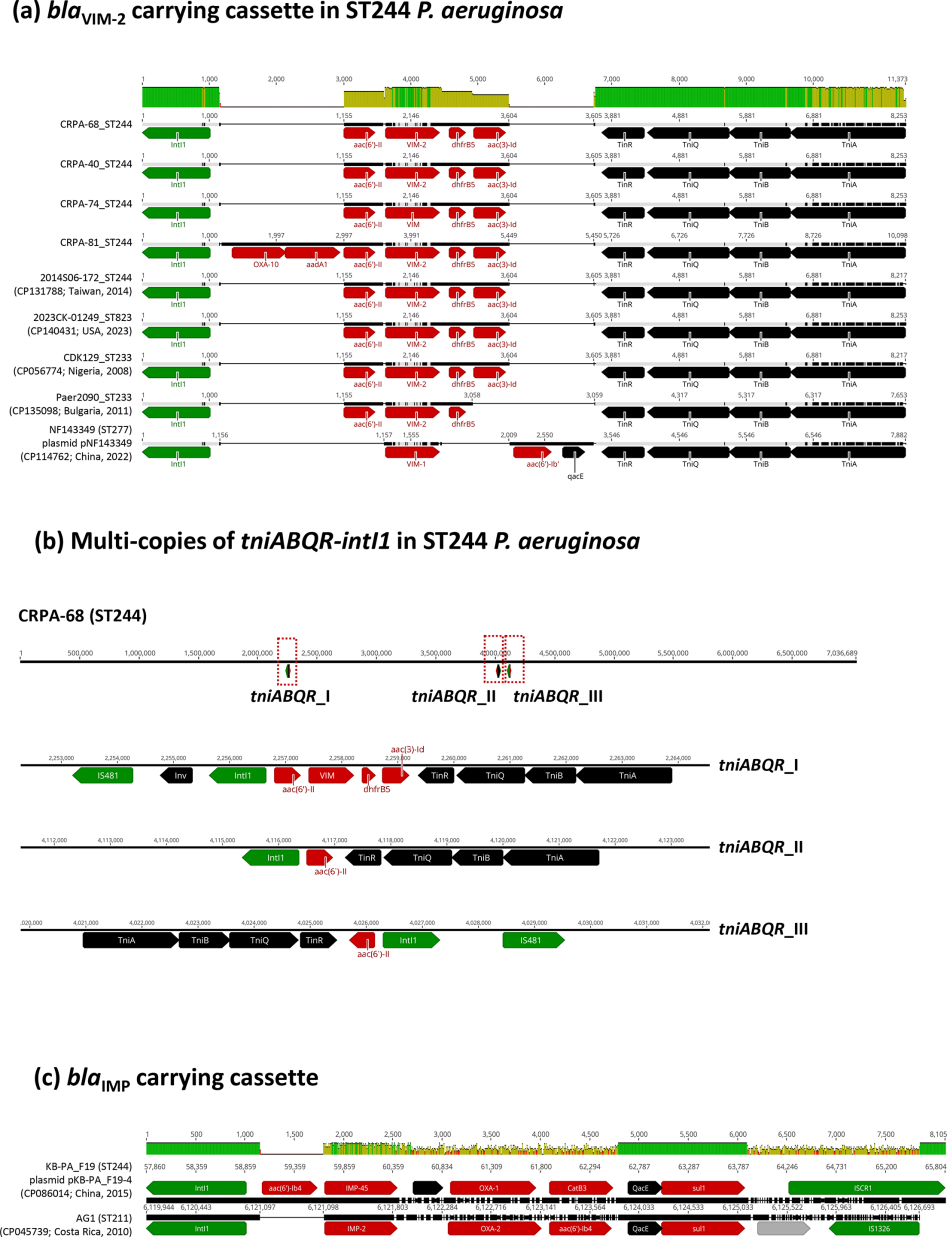
Acquisition of *bla*_VIM-2_ by ST244 CRPA isolates. (**a**) Integration of a *bla*_VIM_-containing cassette mediated by the TniABQR-IntI1 transposition module across various *P. aeruginosa* STs. (**b**) Identification of three chromosomal copies of TniABQR-IntI1 elements carrying AMR genes in the ST244 CRPA isolate CRPA-68. (**c**) *bla*_IMP_-carrying cassette identified in plasmid pF19-4 (CP086014) from the ST244 *P. aeruginosa* KB-PA_F19, isolated in Kunming, China.

VIM (Verona Integron-encoded Metallo-β-lactamase)-producing CRPA has emerged as a major contributor to carbapenem resistance across various regions, primarily driven by the dissemination of plasmid- and integron-associated mechanisms [6,17, 18]. Although ST244 is a recognized high-risk clone in China and metallo-*β*-lactamase production (MBL) is increasing, there have been no previously documented reports of VIM-producing ST244 from China. In this study, we identified four VIM-producing CRPA isolates, all belonging to ST244. The acquisition of *bla*_VIM-2_ was mediated by a mobilizable Tn*402*-like class 1 integron (*tniABQR-intI1*) with the carriage of *aac(6’)-II-bla*_VIM-2_-*dhfrB5-aac(3)-Id*. This Tn*402*-mediated VIM cassette was also identified in a previously sequenced ST244 isolate from Taiwan (2014S06-172, CP131788), as well as in several unrelated STs, including two ST233 isolates from Nigeria (CDK129, CP056774, 2008) and Bulgaria (Pae2090, CP135098, 2011), an ST823 isolate from the USA (2023CK-01249, CP140431, 2023) and an ST277 isolate from China harbouring a related cassette with *bla*_VIM-1_-*aac(6’)-Ib’* instead of *bla*_VIM-2_ (Fig. 8a). The detection of this conserved cassette across diverse lineages and time periods suggests that *bla*_VIM-2_ acquisition likely occurred within the already established ST244 clone via horizontal gene transfer, rather than preceding its initial clonal divergence.

One of the four ST244 isolates in our study, CRPA-81, also harboured *aadA1* and *bla*_OXA-10_, which conferred resistance to amikacin and contributed to elevated imipenem MICs. The complete *tniABQR* transposition module, coupled with a class 1 integron, was found in multiple copies exclusively among ST244 isolates from Taiwan. Up to three copies of *tniABQR-intI1* elements were identified in the genome of CRPA-68 (Fig. 8b). The duplication of AMR genes carried within these elements, such as *aac(6’)-II*, likely contributed to the enhanced resistance phenotypes observed in these ST244 strains. Although complete *tniABQR* modules were absent in ST244 genomes from China, a class 1 integron-mediated resistance cassette *aac(6’)-Ib4-bla_IMP-45_-bla_OXA-1_-catB3-sul1* was detected on a plasmid in an ST244 isolate from Kunming (KB-PA_F19, CP086014) (Fig. 8c). Together, these findings highlight the genomic adaptability of the ST244 lineage to acquire diverse AMR determinants, further consolidating its status as a high-risk clone that can harbour MBL-mediated carbapenem resistance.

## Discussion

A concerning emergence of CRPA was noted from our active surveillance in the clinical setting. Although overall CRPA detection rates (9.5%–19.8%) remained slightly below the national average of over 20%, a marked surge in cases during the first and second quarters of 2022 indicated a potential shift in the local epidemiological landscape. We conducted a phylogenetic analysis on 92 non-duplicate CRPA isolates. We selected 43 representative strains for WGS and phylogenomic characterization to investigate whether the dissemination of high-risk clones contributed to this increase.

Two dominant high-risk lineages were identified: ST292 and ST235*. The latter, ST235*, is a single-locus variant of the globally disseminated ST235 clone, characterized by ExoU production, multidrug resistance and the capacity to acquire carbapenemase genes. In our study, ST235* strains exhibited an even broader AMR profile than typical ST235 isolates (Fig. 4). All ST235* genomes harboured a premature stop codon in *nalD*, a transcriptional repressor of the MexAB-OprM efflux system. Although expression levels were not quantified, *nalD* mutations are an established mechanism of multidrug resistance in *P. aeruginosa* known to derepress MexAB-OprM [19,20]. Therefore, we infer that this loss-of-function mutation likely contributes to the reduced susceptibility to *β*-lactams, fluoroquinolones and aztreonam observed in these strains. Prior exposure to meropenem has been linked to *oprD* mutations and *mexB* overexpression, potentially mediated through regulators such as *nalD*, in hospitalized patients with CRPA infections [21]. Consistently, most of the patients in our cohort from whom ST235* strains were isolated had prior carbapenem exposure (Fig. 2). Additionally, two ST235* isolates carried a *bla*_KPC-2_–*bla*_TEM-1_-containing hybrid cassette, likely acquired from *E. hormaechei* via IS*26*-mediated horizontal gene transfer (Fig. 6). This cassette reinforces the alarming evolutionary capacity of high-risk lineages, such as ST235, to expand their resistance arsenal through interspecies gene exchange, posing a threat to infection control.

Although not as globally widespread as ST235, ST292 has recently emerged as a significant clone implicated in nosocomial outbreaks in China. Notably, ST292 was the most prevalent ST among CRPA isolates collected between 2014 and 2015 in a burn unit at a hospital in Kunming [15], and a broader national surveillance study consistently identified it as the dominant clone in a long-term outbreak of XDR *P. aeruginosa* in the same region [14]. Unlike the earlier Kunming isolates, which were susceptible to ceftazidime, all ST292 strains in our study were resistant to this agent. Genomic analysis revealed that our ST292 strains harboured a class I integron containing the resistance gene cassette *rmtB-tet(G)-floR-sul1-aadA2b-bla_CARB-2_-aac(6’)-Ib3*, as well as a chromosomally integrated *tmexCD-oprJ* efflux system in addition to the intrinsic *mexCD-oprJ* (Fig. 7a). While the ST292 strains lacked carbapenemases, the observed carbapenem resistance was likely mediated by the disruption of the *oprD* gene. Meanwhile, the acquisition of *bla_CARB-2_* broadened *β*-lactam resistance to include ceftazidime, and the presence of *rmtB* and *aac(6’)-Ib3* conferred resistance to amikacin. Notably, three nonsynonymous mutations were identified in the *tmexCD-oprJ* efflux system: an A→G transition in *tmexC* (Trp235Ala), a C→T transversion in *tmexD* (Δ1–117) and a C→G transition in *toprJ* (Ala235Gly). The N-terminal deletion in tMexD3 likely disrupted a transmembrane domain and part of an extracellular motif (Fig. S5), which might alter the structural integrity and function of the efflux system. This alteration could affect the multidrug resistance phenotype of ST292 and further enhance the adaptive potential of this emerging lineage.

The acquisition of the *bla*_VIM-2_-containing cassette in ST244 CRPA isolates from this study was mediated by a mobilizable Tn*402*-like class 1 integron carrying a complete *tniABQR* transposition module. Similar integron structures have been identified in clinical isolates of the *bla*_VIM-2_-producing carbapenem-resistant *Pseudomonas putida* group, supporting their role in horizontal gene transfer among non-fermenting Gram-negative pathogens [22]. Transposition of Tn*402* occurs through cointegrate formation mediated by *tniA*, *tniB* and *tniQ*, followed by site-specific resolution at the *res* region by the *tniR* gene, facilitating the mobilization and chromosomal integration of class 1 integrons [23]. The *bla*_VIM-2_-carrying Tn*402*-like integron identified in our ST244 isolates was also detected in other ST244 strains in Taiwan, as well as in *P. aeruginosa* isolates from different STs, such as ST233, ST823 and ST277, in geographically distinct countries (Fig. 8). While insertion elements such as IS*Pst9*, Tn*7* and IS*6100* have been shown to occur in multiple chromosomal copies in *Pseudomonas stutzeri* and *P. putida* [24,25], previously reported Tn*402*-like integrons have typically been observed as single-copy transposon units. Strikingly, in our study, VIM-producing ST244 CRPA isolates harboured multiple chromosomal copies of *tniABQR-intI1* elements, each carrying AMR genes. This Tn*402*-driven resistance amplification represents a novel and potentially significant evolutionary adaptation in clinical CRPA strains. The genomic plasticity of ST244 emphasizes the importance of genomic surveillance in tracking the emergence and spread of such complex resistance architectures.

*P. aeruginosa* is a genetically diverse opportunistic pathogen. In clinical settings, the extensive use of antibiotics has created a selective advantage, enabling specific CRPA lineages to evolve into high-risk dominant populations with extensive resistance beyond carbapenems. In Taiwan, the prevalence of CRPA has increased annually across healthcare systems. However, knowledge of the transmission dynamics of high-risk lineages remains limited. We acknowledge that the single-centre design and the 14-month study period (July 2021 to September 2022) limit direct extrapolation of our findings to the national level. Nevertheless, as a tertiary referral centre, our institution functions as a sentinel for emerging regional resistance trends. Importantly, ongoing internal surveillance (unpublished data, 2023–2025) suggests that the prevalence of ST292 has remained stable over time, supporting the conclusion that the emergence of this non-carbapenemase-producing yet carbapenem-resistant lineage reflects not a transient fluctuation but the establishment of a persistent high-risk clone. This persistence, together with the identification of other high-risk lineages such as ST235* and VIM-producing ST244, highlights the critical need for continuous genomic surveillance. Such proactive monitoring will be essential to inform national infection control strategies and to mitigate the escalating threat posed by CRPA within healthcare systems.

## Supplementary material

10.1099/mgen.0.001694 Uncited Supplementary Material 1.

## Abbreviations

AMR: antimicrobial resistance
AST: antimicrobial susceptibility testing
CARD: Comprehensive Antibiotic Resistance Database
cgSNP: core-genome SNP
CIPA: carbapenem-intermediate *Pseudomonas aeruginosa*
CLSI: Clinical and Laboratory Standards Institute
CRPA: carbapenem-resistant *Pseudomonas aeruginosa*
CSPA: carbapenem-susceptible *Pseudomonas aeruginosa*
DTR: difficult-to-treat
ICUs: intensive care units
MBL: metallo-*β*-lactamase production
RND: resistance-nodulation-cell division
ST: sequence type
WGS: whole-genome sequencing
XDR: extensively drug-resistant
