# Supplementary material for: Emergence of outbreak-driving high-risk Pseudomonas aeruginosa lineages in Taiwan: phylogenomic insights into ST292 and the ST235* sublineage
Source: Microb Genom. 2026 Apr 21;12(4):001694. doi: 10.1099/mgen.0.001694 (PMC13098761; doi:10.1099/mgen.0.001694)
Supplement: Uncited Supplementary Material 1. [file mgen-12-01694-s001.pdf]

Supplementary Table 1. Accession numbers for the *P. aeruginosa* strains analyzed in this study.

BioProject: PRJNA1266355

Strain No.	BioSample	SRA	Assembly
**CIPA_04**	SAMN48673879	SRS25114418	GCA_050908015.1
**CRPA_07**	SAMN48673881	SRS25114430	GCA_050881575.1
**CRPA_09**	SAMN48673882	SRS25114441	ND[a]
**CRPA_12**	SAMN48673883	SRS25114452	GCA_050908045.1
**CRPA_15**	SAMN48673884	SRS25114456	GCA_050908035.1
**CRPA_18**	SAMN48673885	SRS25114457	ND[a]
**CRPA_20**	SAMN48673886	SRS25114458	ND[a]
**CSPA_21**	SAMN48673921	SRS25114455	ND[a]
**CRPA_24**	SAMN48673887	SRS25114459	ND[a]
**CRPA_25**	SAMN48673888	SRS25114460	GCA_050908115.1
**CRPA_28**	SAMN48673889	SRS25114420	GCA_050846215.1
**CIPA_30**	SAMN48673880	SRS25114419	GCA_050908025.1
**CRPA_31**	SAMN48673890	SRS25114421	GCA_051122775.1
**CRPA_32**	SAMN48673891	SRS25114422	GCA_050846235.1
**CRPA_34**	SAMN48673892	SRS25114423	GCA_050896495.1
**CRPA_35**	SAMN48673893	SRS25114424	GCA_050920805.1
**CRPA_39**	SAMN48673894	SRS25114425	GCA_051122835.1
**CRPA_40**	SAMN48673895	SRS25114426	GCA_050896515.1
**CRPA_44**	SAMN48673896	SRS25114427	GCA_050751225.1
**CRPA_45**	SAMN48673897	SRS25114428	GCA_050908165.1
**CRPA_46**	SAMN48673898	SRS25114429	GCA_051122845.1
**CRPA_49**	SAMN48673899	SRS25114431	GCA_051122785.1
**CRPA_51**	SAMN48673900	SRS25114432	GCA_051122855.1
**CRPA_54**	SAMN48673901	SRS25114433	GCA_051122945.1
**CRPA_55**	SAMN48673902	SRS25114434	GCA_051122955.1
**CRPA_56**	SAMN48673903	SRS25114435	GCA_051122795.1
**CRPA_57**	SAMN48673904	SRS25114436	GCA_050751285.1
**CRPA_58**	SAMN48673905	SRS25114437	GCA_050751345.1
**CRPA_60**	SAMN48673906	SRS25114438	GCA_050751305.1
**CRPA_64**	SAMN48673907	SRS25114439	GCA_051122965.1
**CRPA_68**	SAMN48673908	SRS25114440	GCA_051122805.1
**CRPA_74**	SAMN48673909	SRS25114442	GCA_051122815.1
**CRPA_75**	SAMN48673910	SRS25114443	GCA_050751365.1
**CRPA_78**	SAMN48673911	SRS25114444	GCA_050753205.1
**CRPA_79**	SAMN48673912	SRS25114445	GCA_051122825.1
**CRPA_80**	SAMN48673913	SRS25114446	GCA_051123615.1
**CRPA_81**	SAMN48673914	SRS25114447	GCA_051123625.1
**CRPA_82**	SAMN48673915	SRS25114448	GCA_050896275.1
**CRPA_84**	SAMN48673916	SRS25114449	GCA_051162675.1
**CRPA_91**	SAMN48673917	SRS25114450	GCA_050858815.1
**CRPA_92**	SAMN48673918	SRS25114451	ND[a]
**CRPA_96**	SAMN48673919	SRS25114453	ND[a]
**CRPA_97**	SAMN48673920	SRS25114454	GCA_050858565.1

ND[a]: MinION sequencing was not performed. Only Illumina short-read sequences are available.

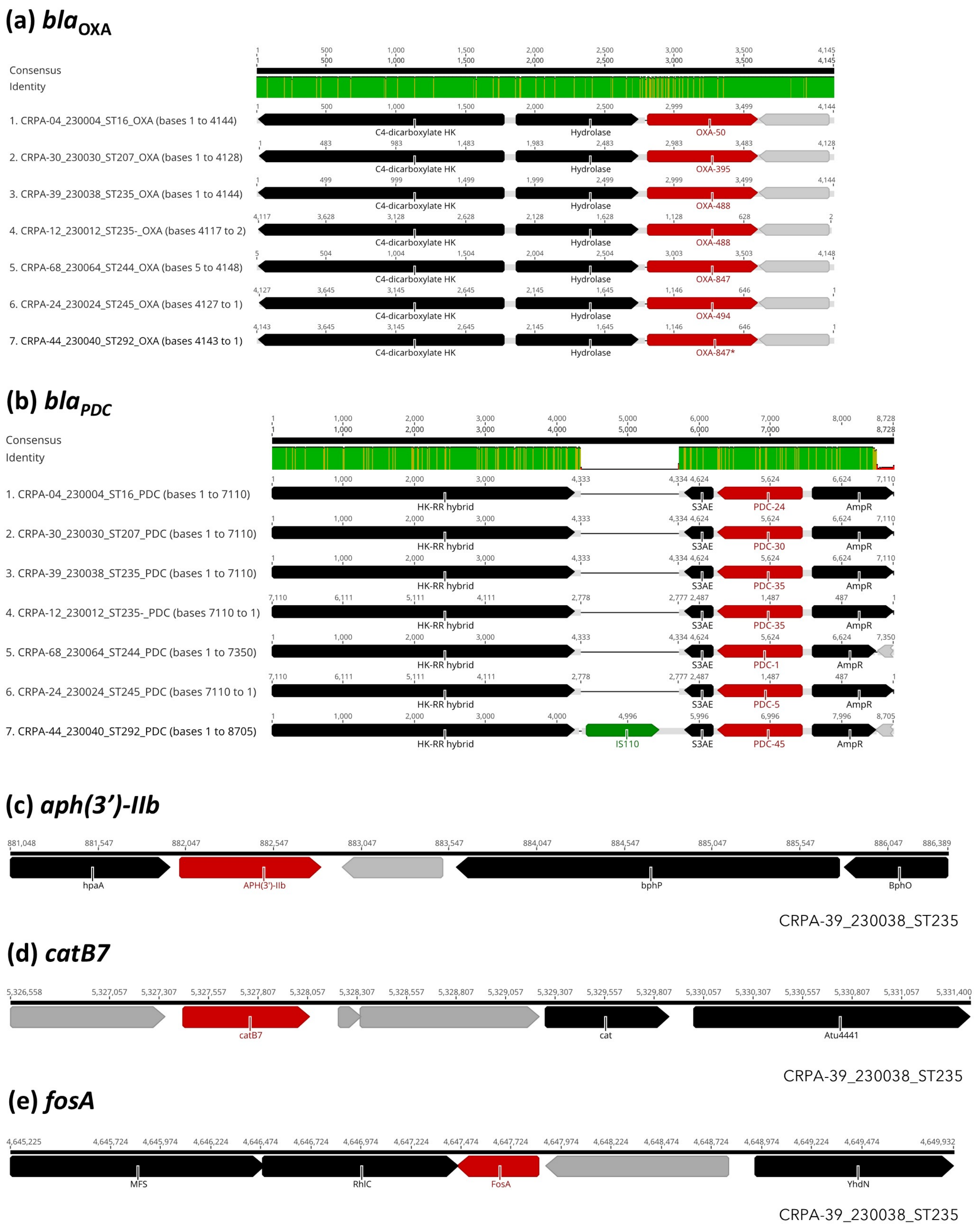

Supplementary Figure 1. Genomic localization of intrinsic resistance genes. Genomic positions of (a) *bla*$_{OXA}$, (b) *bla*$_{PDC}$, (c) *aph(3')-IIb*, (d) *catB7*, and (e) *fosA* in are shown. The subtypes of *bla*$_{OXA}$ and *bla*$_{PDC}$ correlate with specific sequence types (STs), suggesting lineage-associated distribution of these intrinsic β-lactamase genes.

(a) Pairwise alignment of the amino acid sequences of OXA-type β-lactamases

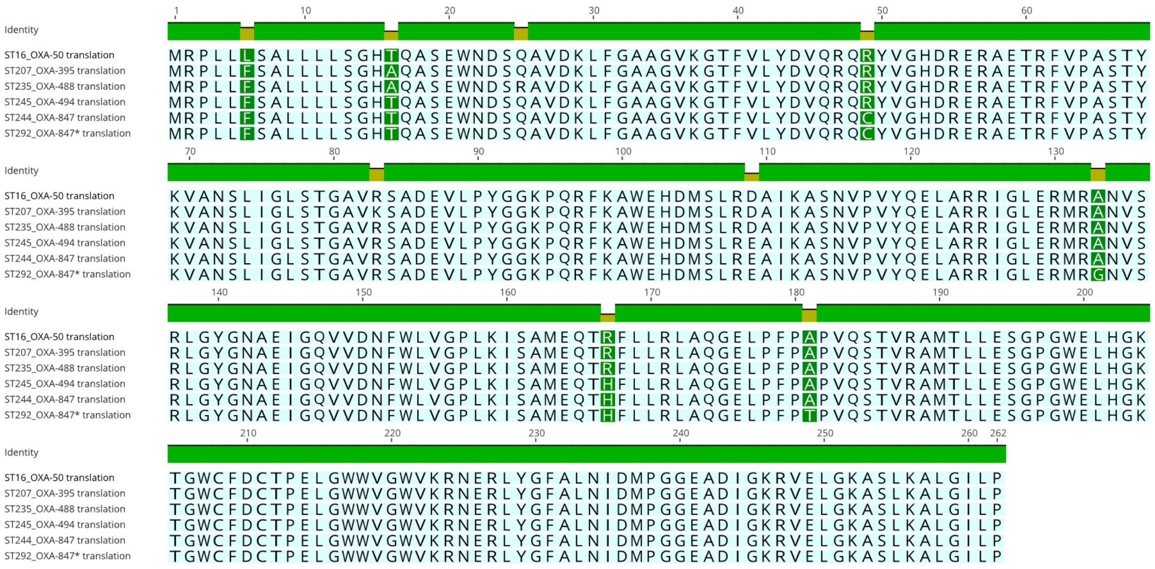

(b) Pairwise alignment of the amino acid sequences of PDC-type β-lactamases

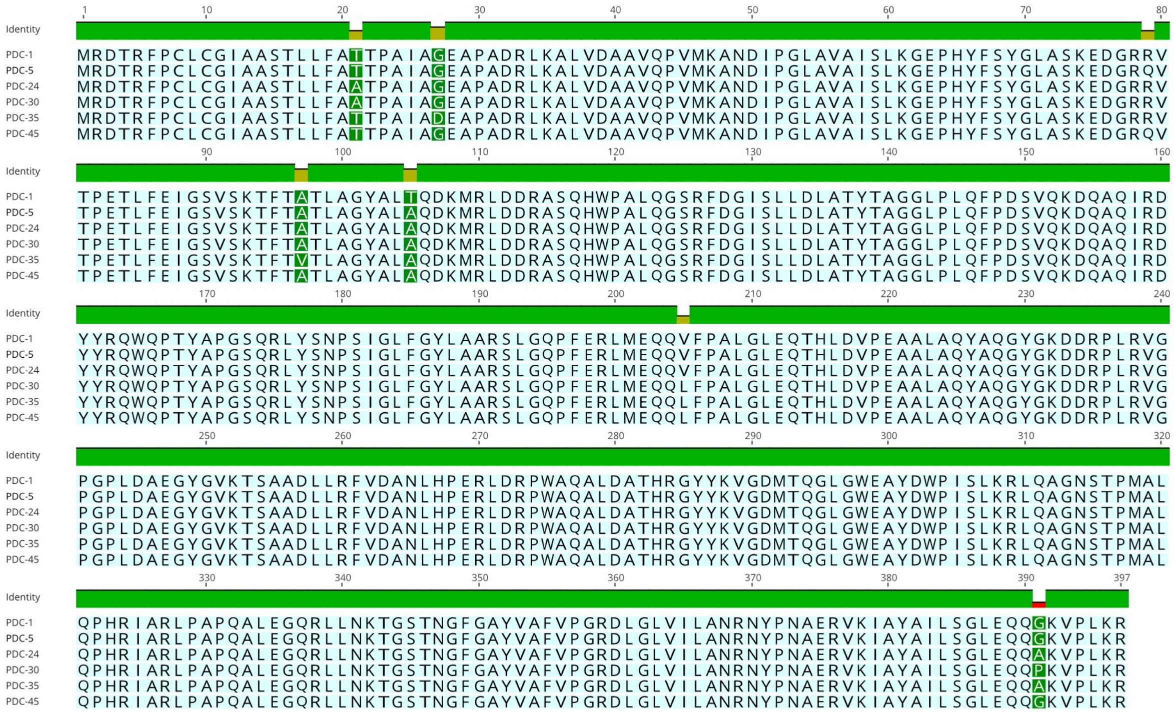

(c) Genomic localization of the orphan porin gene *oprD* in different ST *P. aeruginosa*

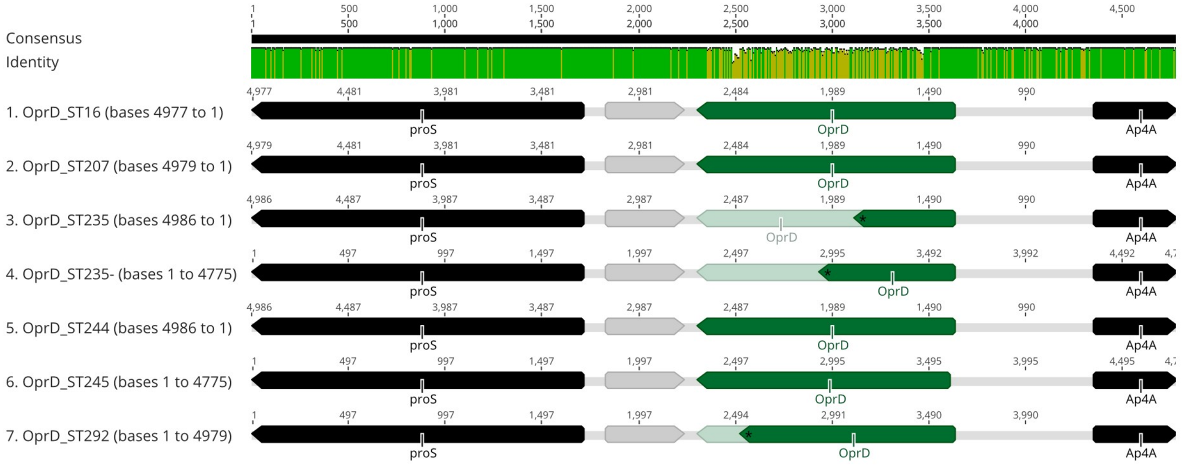

(d) Pairwise alignment of the amino acid sequences of OprD

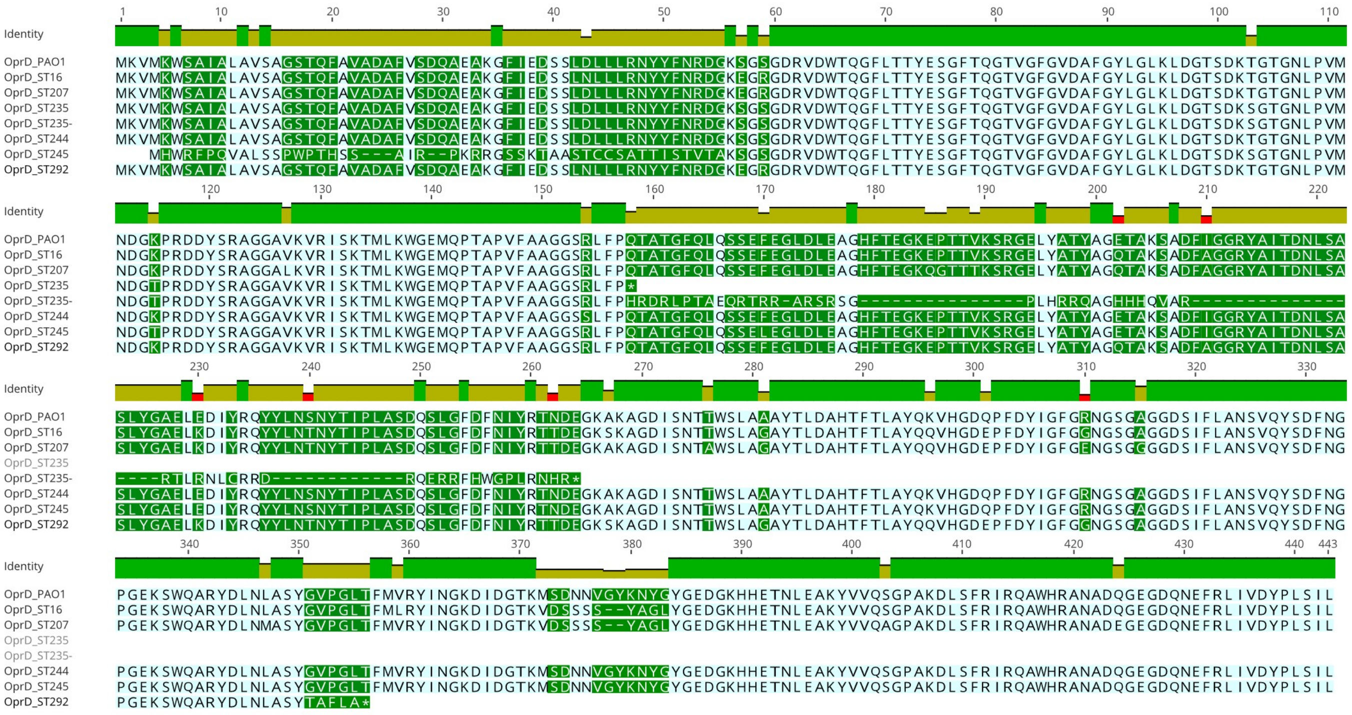

Supplementary Figure 2. Comparative analysis of intrinsic β-lactamase and porin genes in *P. aeruginosa*. (a) Pairwise alignment of amino acid sequences of OXA-type β-lactamases across different STs. (b) Pairwise alignment of PDC-type β-lactamase sequences among diverse STs. (c) Genomic localization of the orphan porin gene *oprD*, with premature stop codons identified in ST292 and ST235* strains. (d) Pairwise alignment of OprD protein sequences from study isolates, compared to the reference OprD protein from *P. aeruginosa* PAO1.

(a) MexRAB-OprM

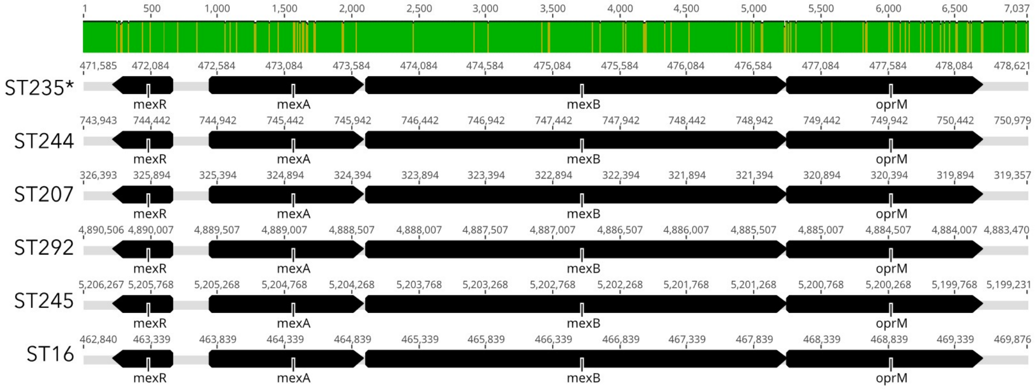

(b) MexCD-OprJ

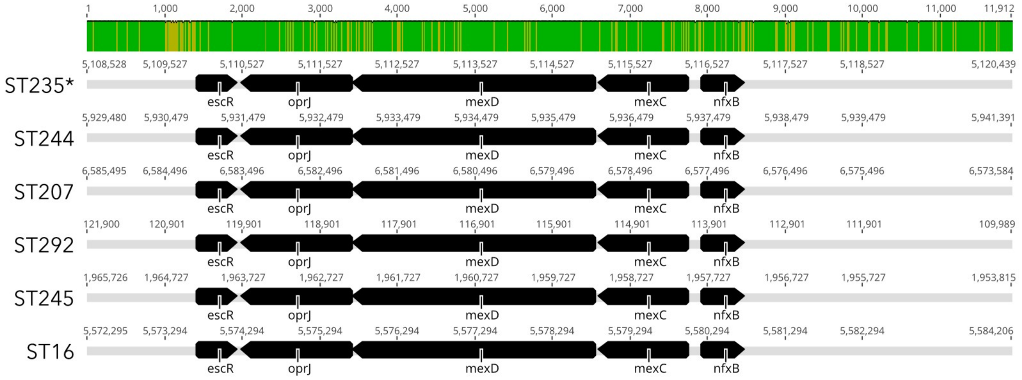

(c) MexEF-OprN

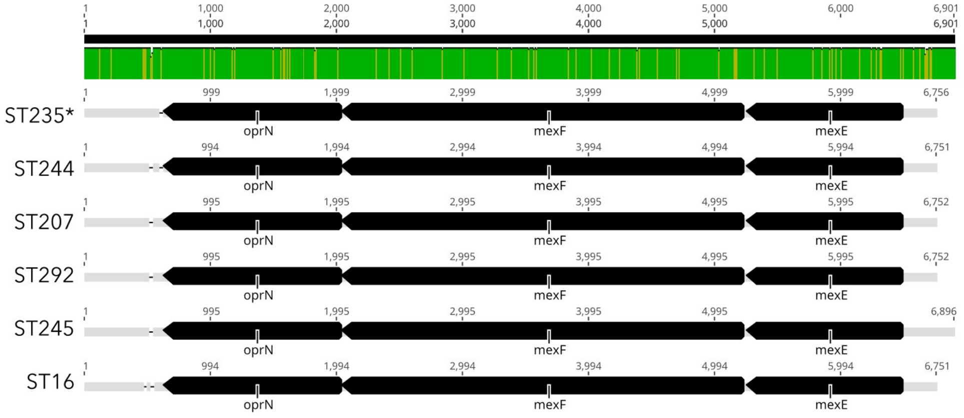

(d) MexGHI-OpmD

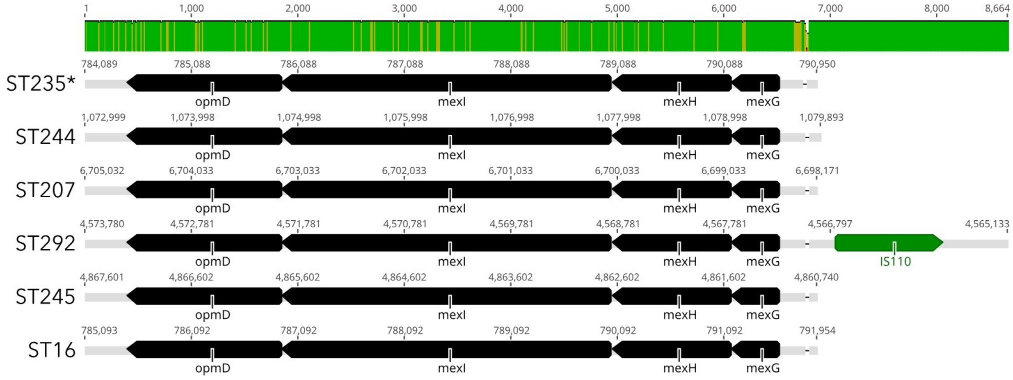

(e) MexJKL

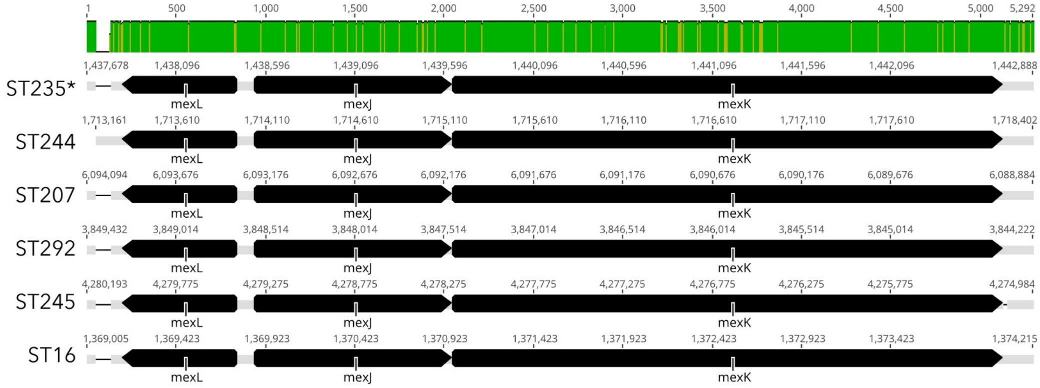

(f) MexMN

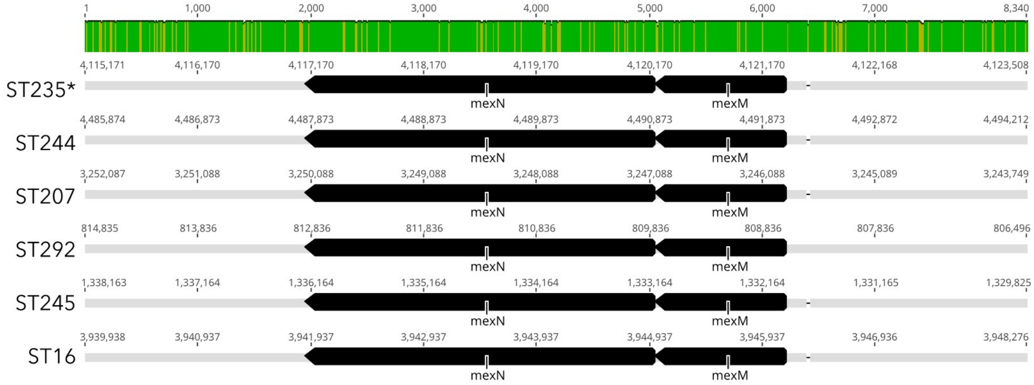

(g) MexPQ-OpmE

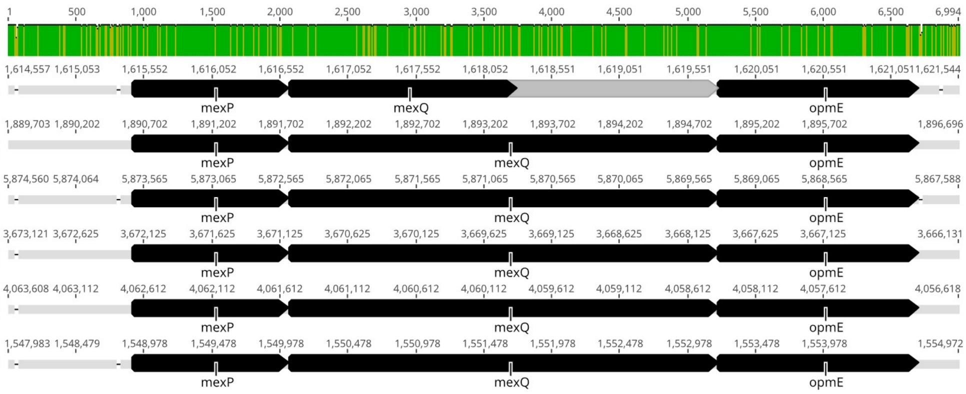

(h) MexVW

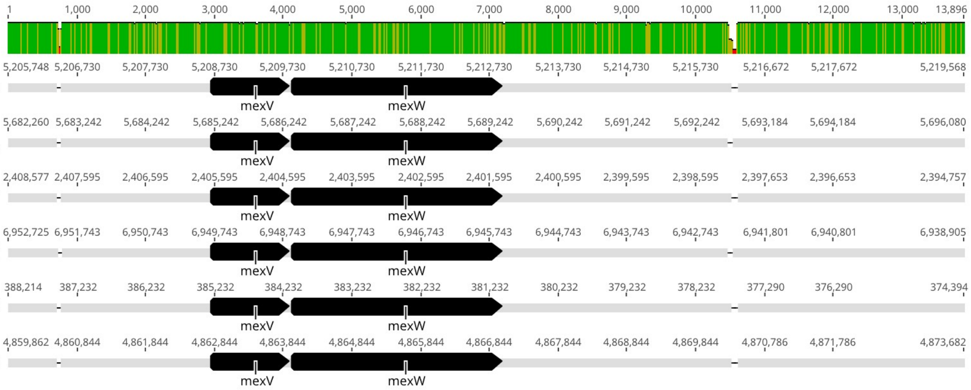

(i) MexXYZ

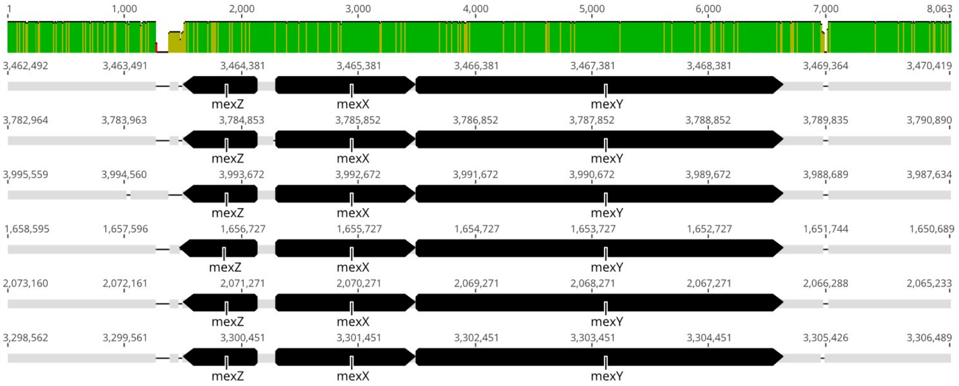

Supplementary Figure 3. Genomic localization and comparative analysis of major RND family efflux systems in *P. aeruginosa*. Genomic positions and structural comparisons of nine major resistance-nodulation-cell division (RND) family efflux systems, (a) MexRAB-OprM, (b) MexCD-OprJ, (c) MexEF-OprN, (d) MexGHI-OpmD, (e) MexJKL, (f) MexMN, (g) MexPQ-OpmE, (h) MexVW, and (i) MexXYZ, are shown across representative *P. aeruginosa* strains from diverse STs.

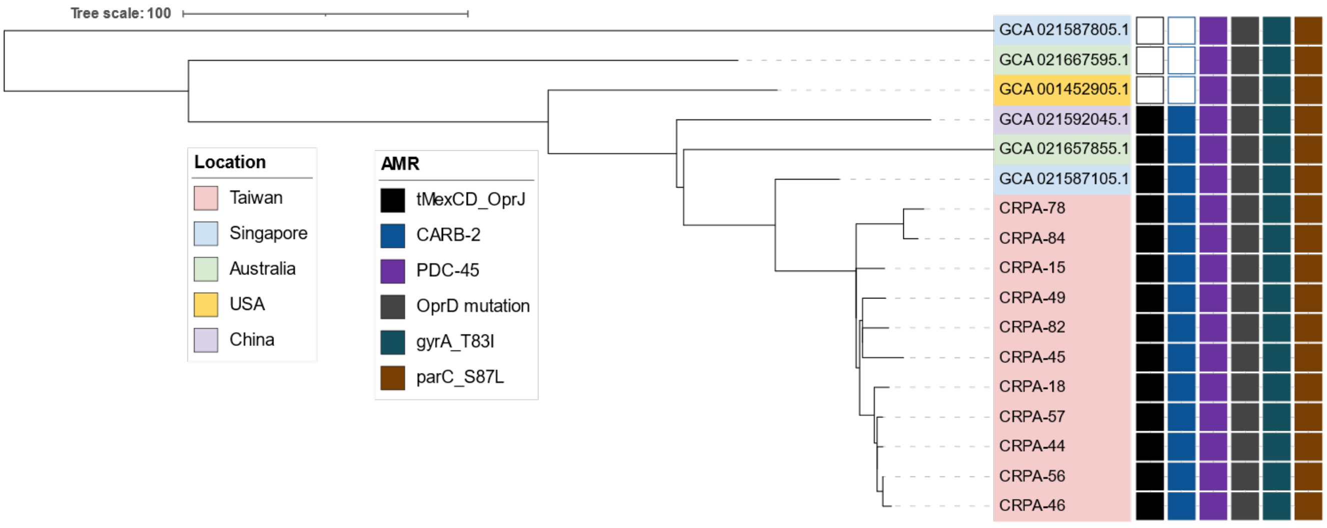

Supplementary Figure 4. Core-genome SNP (cgSNP) phylogenetic analysis of ST292 *P. aeruginosa*. The maximum likelihood phylogenetic tree was constructed using Gubbins *v*3.4.3, which generate a recombination-free phylogeny reflecting true clonal descent. The analysis included 17 ST292 genomes: 11 sequenced in this study and 6 publicly available assemblies downloaded from GenBank. Tree annotation and visualization were performed using iTOL *v*7.4.2. The scale bar represents 100 nucleotide substitutions. Geographical origins are indicated by the color strip adjacent to the sample name: Taiwan (pink), Singapore (light blue), Australia (green), USA (yellow), and China (purple). The presence of key antimicrobial resistance (AMR) determinants is shown in the heatmap to the right: tMexCD_OprJ (black), CARB-2 (dark blue), PDC-45 (purple), OprD mutation (dark grey), gyrA_T83I (dark teal), and parC_S87L (brown). A filled square indicates the presence of the determinant, while an empty square indicates its absence.

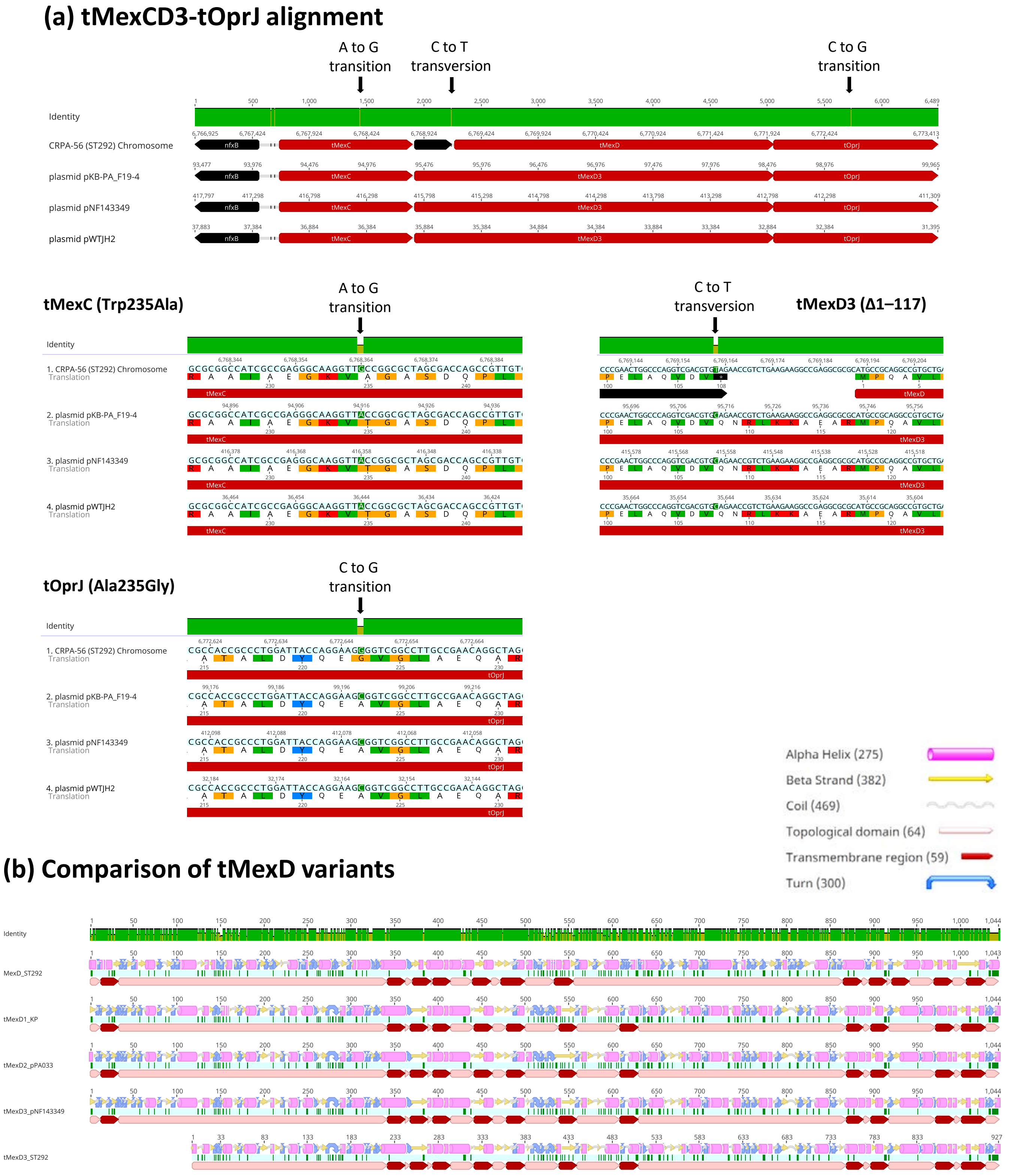

Supplementary Figure 5. Nonsynonymous mutations in the *tMexCD-tOprJ* efflux system of ST292 strains. (a) Identified point mutations include an A-to-G transition in *tmexC*, a C-to-T transversion in *tmexD*, and a C-to-G transition in *toprJ*, resulting in amino acid changes: tMexC (Trp235Ala), tMexD3 (Δ1-117), and tOprJ (Ala235Gly), respectively. (b) Predicted secondary structure of tMexD3 indicates that the N-terminal deletion (Δ1-117) in the variant of ST292 strains likely disrupts a transmembrane domain and part of an extracellular motif.
